# An EGFR and AKT Signaling Pathway was Identified with Mediation Model in Osteosarcomas Clinical Study

**Published:** 2007-12-11

**Authors:** Huiyun Wu, Nicole E. Muscato, Adriana Gonzalez, Yu Shyr

**Affiliations:** 1 Department of Biostatistics; 2 Department of Pathology, Vanderbilt University Medical Center, Nashville, TN 37232-6848, U.S.A

**Keywords:** mediation model, EGFR, AKT, osteosarcomas, clinical study

## Abstract

Identification of correlation pattern and signal pathway among biomarkers in patients has become increasingly interesting for its potential values in diagnosis, treatment and prognosis. EGFR and p-AKT signaling in osteosarcoma (OS) patients were analyzed for its relationship with cancer cell proliferation maker, Ki-67, using causal procedures and statistical tests. A total of 69 patients were collected who present to Vanderbilt University Medical Center with newly diagnosed, previously untreated osteosarcomas during the clinical study period 1994 through 2003. Tissue microarrays were constructed for EGFR, p-AKT and Ki-67. The mediation model was constructed with structural equation model (SEM) for the causal analysis of the three biomarkers in osteosarcoma patients. The results suggested a mediating effect of p-AKT for the causal relationship between EGFR and Ki-67. The study also found significant associations between EGFR and Ki-67 (p = 0.002), EGFR and p-AKT (p = 0.027), and p-AKT and Ki-67 controlling EGFR (p = 0.004). After the impact of EGFR on Ki-67 was accounted for by p-AKT, the relation between EGFR and Ki-67 was no longer significant (p = 0.381). The mediating effect was confirmed with Sobel test (p < 0.001) and Goodman (I) test (p < 0.001). The study indicated that a mediation model could be an approach to exploring the correlation pattern of EGFR and AKT signal pathway for cancer cell proliferation in OS patients in clinical study.

## Introduction

Osteosarcoma (OS) is the most common primary bone sarcoma with approximately 900 new cases in the United States each year. Although chemotherapy and surgery have improved survival, 5-year survival has reached a plateau of approximately 70% and this figure has not changed significantly for almost 20 years (Ferguson and Goorin, 2001). New treatments, especially those that may target specific cellular pathway components, are of particular interest (Anninga et al. 2004; Hughes et al. 2004). Among these components, epidermal growth factor receptor (EGFR), a membrane protein, has been well studied for its function in cancer biology. EGFR has been found to be expressed in a variety of human tumors of epithelial origin including lung, breast, colorectal, pancreatic, ovarian (Arteaga, 2002; Tabernero et al. 2004) as well as OS (Dobashi et al. 2004; Hughes et al. 2004). Activation of EGFR causes increased cell proliferation, decreased apoptosis, and enhanced tumor cell motility and neo-angiogenesis. All these events are mediated by a sequence of proteins including phosphatidylinositol-3 kinase (PI3K) and protein kinase B (AKT). AKT acts downstream of PI3K to regulate many biological processes, ultimately resulting in a cascade of cellular responses and events such as survival, proliferation, and cell growth (Tabernero et al. 2004; Vivanco and Sawyers, 2002; Xia et al. 2002). Studies have already shown that the inhibition of AKT blocked the EGFR-initiated transformation of the cells (Lan et al. 2000; Vivanco and Sawyers, 2002). However, studies of the association between EGFR and cancer development in OS patients are few. Previous studies of the role of EGFR in OS involved relatively small numbers of cases (Dobashi et al. 2004; Hughes et al. 2004). In addition, no reports are available on the correlation pattern among EGFR, AKT, and Ki-67, a cancer cell proliferation index.

Mediation model may be an approach to looking at the correlation pattern among the biomarkers with its causal hypothesis (MacKinnon et al. 1995; MacKinnon, 1993). Usually, bi-variate correlation models can be applied to address the relationship between an explanatory variable and an outcome. But these models are unlikely to describe adequately the nature of the relationships among the related variables, such as the three biomarkers in the present study. An alternative approach is to use more complex path analysis models such as mediation model. These more complex explanatory models could yield important information about the nature of the relationships (Beck, 2004). Mediation model was designed to analyze a causal hypothesis whereby an independent variable causes a mediator which causes a dependent variable. A variable may be considered a mediator (*M*) to the extent to which it carries the influence of a given independent variable (*X*) to a given dependent variable (*Y*). Mediation can be said to occur when (1) *X* significantly affects *M*, (2) *X* significantly affects *Y* in the absence of *M*, (3) *M* has a significant effect on *Y* in the presence of *X*, and (4) the effect of *X* on *Y* shrinks upon the inclusion of the *M* in the model. The mediation effect can also be tested with some appropriate statistical procedures (MacKinnon et al. 1995; MacKinnon, 1993).

The focus of the present study is to answer whether the expression of EGFR and AKT correlated with the cancer cell proliferation and how the three biomarkers were correlated in OS patients by using a mediation model with an interest in exploring its value in treatment, diagnosis and prognosis.

## Materials and Methods

### Patients

Patients were identified by searching through the pathology database which contained pathological results for those who present to Vanderbilt University Medical Center from 1994 through 2003 with newly diagnosed, previously untreated OS. The Institutional Review Board of Vanderbilt Medical School reviewed and approved the study. All the patients completed one course of chemotherapy prior to resection of their tumors. The informed consent was waived. Slides and archival tissue blocks from these patients’ resections were retrieved. Two pathologists experienced in the diagnosis of bone tumors reviewed all the diagnostic slides and classified these tumors according to the World Health Organization (WHO) classifi-cation of tumors. One hundred OS patients reviewed were categorized as conventional OS including the three main histologic subtypes of conventional OS: osteoblastic, chondroblastic, and fibroblastic subtypes. Excluded in the analysis were other rare osteosarcoma subtypes including small cell OS, parosteal OS, periosteal OS, high grade surface OS, NOS, and low grade intramedullary OS, resulting in a total of 69 subjects for the analysis. Clinical data gathered for these patients included date of biopsy or resection, presence of local recurrence, presence of metastasis, and last date of follow-up. Demographic information of the patients was also collected in this study.

### Tissue microarray and immunostaining

Three tissue microarrays (TMAs) were constructed from one representative 1mm punch taken from paraffin-embedded tissue blocks of each tumor. Paraffin immunoperoxidase studies were performed with a monoclonal antibody against total EGFR (Zymed, prediluted antibody, Cat. No. 08-4205) and a monoclonal antibody against phos-phorylated AKT (p-AKT, Cell Signaling). All TMAs were immunostained for the proliferation marker Ki-67 (Dako, MIB-1).

For EGFR and p-AKT, expression was scored as index (index = intensity of staining × % tumor cells staining). Ki-67 nuclear immunostaining was evaluated as an estimated percentage of positive tumor cells. Membrane staining was evaluated for EGFR whereas both nuclear and cytoplasmic stains were noted for p-AKT. In statistical analysis, we used nuclear score of p-AKT.

### Statistical analysis

**Figure 1:**
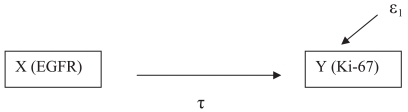
Path for Model 1. A two-variable correlation model between an explanatory variable and an outcome variable. τ is the parameter of interest. ɛ is the error term.

Two approaches were taken to analyze mediating effect; one was causal analysis and the other was statistical test. For causal analysis, we built three models to identify the mediating effect. Model (1) is illustrated by Figure 1 in which we look for a significant association between *X* and *Y*, i.e. (τ).

(1) $$Y=\beta 0(1)+\tau X+{ɛ}_{1}$$

**Figure 2:**
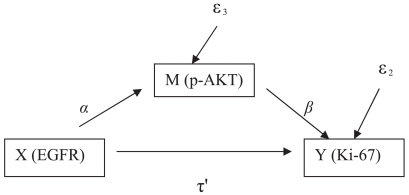
Paths for Model 2 and Model 3. A three-variable mediation model. X, M, and Y are symbols of the three variables. α*,* β and τ′ are the parameters of interest. ɛ is the error term.

Model (2) and (3) are illustrated by Figure 2 in which the effect of *X* on *Y* goes through *M*. The parameter *α* in Model (3) relates *X* to *M*, whereas parameter *β* in Model (2) relates the mediator to the outcome. In these two models we look for significant *α* and *β*, and insignificant τ′.

(2) $$Y=\beta 0(2)+\tau^{\prime }X+\beta M+{ɛ}_{2}$$

(3) $$M=\beta 0(3)+\alpha X+{ɛ}_{3}$$

From the three models, we have (MacKinnon et al. 2000):

Direct effect = τ′

Mediated effect = *α β*

Total effect = τ

For the statistical tests, we ran Sobel test (Sobel, 1982), which basically tests the null hypothesis that the indirect effect (product of *α* and *β* ) is zero in the population from which the sample data are randomly drawn. The test statistics is computed by dividing the indirect coefficient by its standard error.

Sobel test:

$$\text{Z}-\text{value}=\alpha *\beta /\sqrt{\alpha^{2}\sigma_{\beta }^{2}+\beta^{2}\sigma_{\alpha }^{2}}$$

Goodman (I) test:

$$\text{Z}-\text{value}=\alpha *\beta /\sqrt{\alpha^{2}\sigma_{\beta }^{2}+\beta^{2}\sigma_{\alpha }^{2}+\alpha_{\alpha }^{2}\sigma_{\beta }^{2}}$$

We also performed Goodman (I) version of Sobel test according to the recommendation (MacKinnon et al. 1995). The test has slightly different standard error term and it also avoids unnecessary assumption that the product of σ_α_ and σ_β_ is too small.

**Figure 3:**
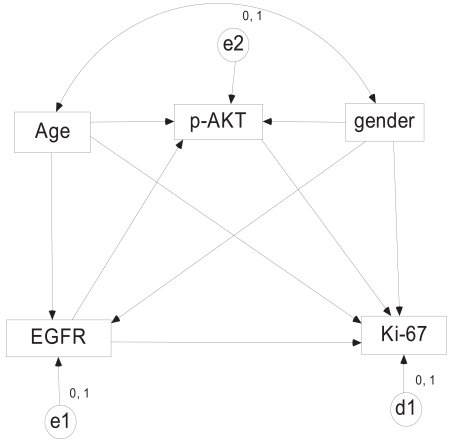
Structural equation modeling for mediation effect. Dependent variable Ki-67 is predicted with single-headed EGFR and p-AKT. Each single-headed arrow represents a regression weights. Age and gender are considered to be associated with all the three biomarkers and are also allowed to be correlated and represented with double-headed arrow. Circles of e1, e2 and d1 are error terms with mean at 0 and variance at 1.

The three causal models were constructed and implemented with structural equation model (SEM), which computed the three models simultaneously. The SEM also adjusted for age and sex (Fig. 3). A bootstrap procedure was incorporated to the computations considering the small sample size and non-normal data distribution as recommended (MacKinnon, 2004; Shrout and Bolger, 2002). In addition to Sobel test and Goodman test with formula developed by Mackinnon and Dwyer (MacKinnon et al. 1995; MacKinnon, 1993), the mediating effect was also tested using spline regression that allows for flexible relationship among the three variables. In our study, we used 3 knots in spline regression model for the small sample size, which generated two degree of freedom test with two coefficients (two *α*s and two *β*s). Instead of performing multiple tests for α*β, we ran the test for a mediation of overall effects of α and overall effects of *β* by using F statistics. To have a general view of the data distribution, three scatter-plots were made for predictive biomarker and dependent biomarker. The plots were also overlapped with curves estimated using a nonpara-metric smoother. The package of AMOS 5.0 (SPSS, Chicago, IL) was used for mediation modeling and R version 2.2.0 ((http://cran.r-project.org/) for the spline regression. The interactive calculation tool at http://www.unc.edu/~preacher/sobel/sobel.htm was employed for Sobel test and Goodman test. All the p values were two-tailed with statistical significance at p < 0.05.

## Results

**Figure 4:**
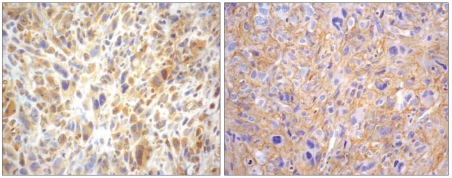
Expression of EGFR and p-AKT. Left panel: membrane staining in tumor cells with EGFR; right panel: nuclear and cytoplasmic staining in tumor cells with p-AKT.

**Figure 5:**
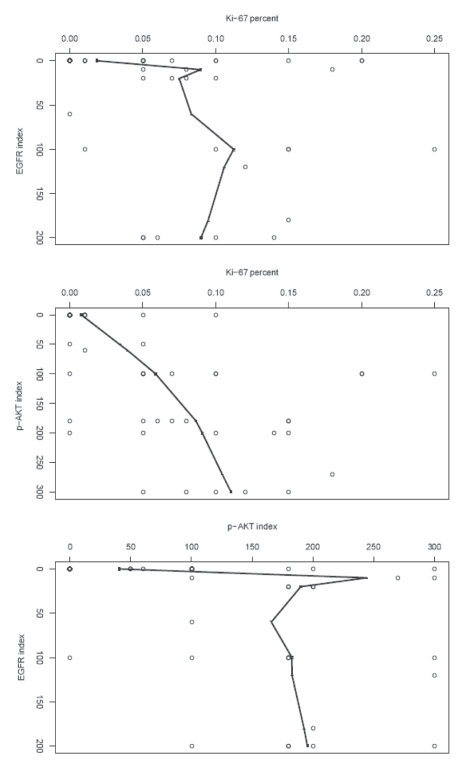
Scatter plots with lowess lines. Circles are data points and lines are the lowess smoothing lines.

The immunostaining of EGFR and p-AKT were shown in Figure 4. The characteristic of the patients were listed in Table 1. The age distribution in OS patients was bi-model, one occurring in the second decade of life and the other over forty with an average of 29 years. The patients were 47% female. The major tumor subtype was osteoblastic (71%). The marginal relations between predictive bio-marker and dependent biomarker indicated a positive trend for all the three relationships with the one between p-AKT and Ki-67 more linear (Fig. 5).

The results from the 3 causal models were summarized in Table 2. A significant association was found between EGFR and Ki-67(p = 0.002,τ). EGFR was a significant predictor for p-AKT (p = 0.027, *α*), which in turn was a significant predictor for Ki-67 controlling EGFR (p = 0.004, *β* ). After p-AKT was taken into account, the relation between EGFR and Ki-67 was no longer significant (p = 0.381, τ′), suggesting a mediating effect of p-AKT on the proposed causal relation between EGFR and Ki-67 in OS patients. As detailed in Table 2, the total effect of EGFR on Ki-67 was 0.038, and the mediated effect of 0.029 accounted for 76% of the total effect.

Sobel test and Goodman (I) test confirmed a significant indirect effect of α*β (p < 0.001) (Table 3), supporting the results from the causal analysis. The results of the two statistical tests with spline regression remained significant (Table 3).

## Discussion

Correlations among biomarkers and signal pathway analysis have been becoming increasingly interesting in cancer biology and drug research and development. Identification of biomarker correlation pattern and signal pathways has shed light in elucidating the mechanisms of cancer development and provided a new avenue to cancer treatment (Curtis et al. 2005; Mao et al. 2006). However, the available analysis approaches and tools are limited, and no much effort has been made to look for the signal pathways in population study. The concept and application of medication model have been seen in social science, behavioral science, and preventive medicine. Mediation model analyzes “how” an effect occurs by hypothesizing a causal sequence (MacKinnon, 1993). In practice, mediation is the mechanism by which a variable affects another variable. Interestingly, signaling events are ordered both spatially and temporally, and the processes of signal transduction in cells are also in causal steps.

Our study found a significant association between EFGR expression and proliferation marker Ki-67 (τ in Model 1), supporting the findings from biological studies (Anninga et al. 2004; Arteaga, 2002). This result suggested an involvement of EGFR in OS development. In studies of patient survival, EGFR has been identified as a strong prognostic indicator in a wide range of tumors (Gorlick et al. 1999; Magne et al. 2001; Neskovic-Konstantinovic et al. 1999; Tsutsui et al. 2002). Our study also indicated that EGFR was a significant predictor of p-AKT (*α* in Model 3). Biological experiments have demonstrated that the activation of EGFR was the essential upstream event for AKT to function and cause its downstream events (Arteaga, 2002; Lan et al. 2000). According to Model (2), the present study clearly showed that the association between *X* and *Y* was remarkably attenuated and no longer significant after *M* was controlled (τ′) while *M* was also a significant predictor of *Y* in the same model. Translated into biology, the findings said that once p-AKT was introduced in the analysis of the relationship between EGFR and Ki-67, the impact of EGFR on Ki-67 was carried by p-AKT which was also significantly associated with Ki-67. Experiments have been conducted and shown that p-AKT was up-regulated upon the activation of EGFR and it mediated the signal from EGFR to cancer cell proliferation (Lan et al. 2000; Vivanco and Sawyers, 2002). In a clinical study of EGFR tyrosine kinase inhibitor (erlotinib) on malignant gliomas patients, AKT activation status was found to determine the response of the tissue to the inhibitor (Haas-Kogan et al. 2005), suggesting that p-AKT had mediated EGFR signaling in the patients.

In addition to the causal analysis, Sobel test and Goodman test also indicated a mediating effect of p-AKT for EGFR signaling to Ki-67 in the patients. These test results were formal statistical evidence for the mediation (MacKinnon, 1993). Thus, both causal procedures and statistical tests strongly suggested that the associations among EGFR, p-AKT and Ki-67 in the OS patients fell into a mediation pattern.

SEM was used in the study because of its advantage in joint modeling. A concern on using SEM is that it assumes linear relationship that may not be necessarily plausible in biology. In many cases, the property of outcome does not behave linearly in all the predictors (Harrell Jr., 2001). Our study dealt with this issue by using spline regression that allows for nonlinear relationship. Two coefficients were computed in spline regression with knots = 3 in our study. In stead of performing multiple tests for α*β = 0, we tested mediation for overall effects of α and overall effects of *β* by applying *F* statistics to the Sobel test and Goodman test. Similar results were obtained with the spline regression, confirming the mediating effect of p-AKT in the path.

Confounding effect is a common issue in multiple regression analysis with distinguished properties. In concept, mediation is part of the causal chain, whereas confounding is a cause of the outcome and correlated with the independent variable. Confounding differs from mediating in that confounding explains the relationship between *X* and *Y* whereas mediating carries the relationship between *X* and *Y* (Robins, 1989). Confounding does not imply a causal relationship among the three variables, thus there is no indirect effect from *X* to *Y*. Unlike mediating effect, confounding effect can’t be statistically tested (MacKinnon et al. 2000). Interaction is another issue in multivariate analysis. The difference between interaction and mediation is subtle. Interaction test is basically to test the product of the two exposure variables = 0 with relation to the outcome variable using either likelihood ratio test or Wald test whereas mediation test is to test the net effect of α*β = 0 (or *α*−*β* = 0) with Sobel test or other tests. Interaction test tells whether the effect of *X* on *Y* is modified at different levels of the third variable; however, mediation test tells us whether the effect of *X* on *Y* is changed with or without the third variable. In interaction, the two variables are both independent variables. But in mediation, the mediator is both a dependent variable relative to *X* and an independent variable relative to the outcome. Interaction and mediation may coexist, but their exact analytical procedures in this case have not been completely articulated (Muller et al. 2005). Interaction has no causality and thus interaction does not imply mediation.

Although the causal analysis and statistical test suggested a mediated effect of EGFR to Ki-67 by p-AKT, the study was limited by the cross-sectional nature which raised a concern on the causal assumption. Nevertheless, the biological studies that signaling events are ordered temporally and spatially have conditioned our models still in a follow-up pattern, however conceptually. In fact, the mediation model has been used in many other cross-sectional studies (Beck et al. 2005; Frone, 1999; Frye and Garber, 2005). In Beck’s study, mediation analysis indicated that pain influenced fatigue directly as well as indirectly by its effect on sleep (Beck et al. 2005). Recently in a cross- sectional study, Xie et al. demonstrated an association between BMI and depression mediated by perceived peer isolation in adolescents (Xie et al. 2005). Small sample size is another limitation in our study. Hence, bootstrapping was employed for this issue and possible non-normal distribution.

In conclusion, the present study found an association between EGFR and cancer cell proliferation biomarker Ki-67 in OS patients, and this association was carried by the p-AKT. The mediation model explained and confirmed the EGFR and p-AKT pathway and its effect on cancer development in OS patients with reasonable satisfaction. Thus, mediation model may be a useful approach to analyzing pathway information in OS patients. The findings from the study may provide additional information for cancer diagnosis and treatment with specific targets.

## Figures and Tables

**Table 1:** Characteristic of the patients (N = 69).

Characteristic	Measurement
Age (mean ± SD)	28.9 ± 15.6
Female (%)	33 (48%)
Diagnosis (%)	
osteoblastic	49 (71%)
chondroblastic	7 (10%)
fibroblastic	5 (7%)
other	8 (12%)
Recurrence (%)^1^	31 (48)
Metastasis (%)^2^	33 (49)

1 Three patients had missing values.

2 Two patients had missing values.

**Table 2:** Effect table1.

Path	Model	parameter	Direct effects (95% CI)	Indirect effects (95% CI)	Total effect (95% CI)	p value^5^
EGFR → Ki-67	(1)	τ	0.038 (0.018, 0.058)	NA	0.038 (0.018, 0.058)	0.002
EGFR → Ki-67	(2)	τ′	0.009 (−0.012, 0.030)^2^	0.029 (0.014, 0.044)^3^	0.038 (0.018, 0.058)^4^	0.381
p-AKT → Ki-67	(2)	β	0.034 (0.020, 0.048)	NA	0.034 (0.020, 0.048)	0.004
EGFR → p-AKT	(3)	α	0.850 (0.550, 1.150)	NA	0.850 (0.550, 1.150)	0.027

1 Data are expressed as coefficient for effects or P values for parameter tested. NA = not applicable.

2 Direct effects of EGFR → Ki-67 = τ′ = 0.009 is the estimate after adjusting for p-AKT.

3 Indirect effects of EGFR → Ki-67 = α × β = 0.850 × 0.034 = 0.029. Confidence interval was calculated with Sobel standard error.

4 Total effect of EGFR → Ki-67 = α × β + τ′ = 0.850 × 0.034 + 0.009 = 0.038.

5 p values were computed for the parameters.

**Table 3:** Results of statistical tests.

Test	p value with SEM	p value with spline regression
Sobel	<0.001	<0.001
Goodman(I)	<0.001	<0.001
